# Common genetic variants, acting additively, are a major source of risk for autism

**DOI:** 10.1186/2040-2392-3-9

**Published:** 2012-10-15

**Authors:** Lambertus Klei, Stephan J Sanders, Michael T Murtha, Vanessa Hus, Jennifer K Lowe, A Jeremy Willsey, Daniel Moreno-De-Luca, Timothy W Yu, Eric Fombonne, Daniel Geschwind, Dorothy E Grice, David H Ledbetter, Catherine Lord, Shrikant M Mane, Christa Lese Martin, Donna M Martin, Eric M Morrow, Christopher A Walsh, Nadine M Melhem, Pauline Chaste, James S Sutcliffe, Matthew W State, Edwin H Cook, Kathryn Roeder, Bernie Devlin

**Affiliations:** 1 Department of Psychiatry, University of Pittsburgh School of Medicine, Pittsburgh, Pennsylvania, USA; 2 Program on Neurogenetics, Yale University School of Medicine, New Haven, Connecticut, USA; 3 Child Study Center, Yale University School of Medicine, New Haven, Connecticut, USA; 4 Department of Psychiatry, Yale University School of Medicine, New Haven, Connecticut, USA; 5 Department of Genetics, Yale University School of Medicine, New Haven, Connecticut, USA; 6 Department of Psychology, University of Michigan, Ann Arbor, MI, USA; 7 Neurogenetics Program, Department of Neurology and Center for Autism Research and Treatment, Semel Institute, David Geffen School of Medicine, University of California Los Angeles, Los Angeles, California, USA; 8 Department of Human Genetics, Emory University School of Medicine, Atlanta, Georgia, USA; 9 Division of Genetics, Children's Hospital Boston, Harvard Medical School, Boston, Massachusetts, USA; 10 Department of Psychiatry, McGill University, Montreal Children's Hospital, Montreal, QC, H3Z 1P2, Canada; 11 Department of Psychiatry, Mount Sinai School of Medicine, New York, New York, USA; 12 Geisinger Health System, Danville, Pennsylvania, USA; 13 Center for Autism and the Developing Brain, Weill Cornell Medical College, White Plains, New York, USA; 14 Yale Center for Genome Analysis, Orange, Connecticut, USA; 15 Departments of Pediatrics and Human Genetics, The University of Michigan Medical Center, Ann Arbor, Michigan, USA; 16 Department of Molecular Biology, Cell Biology and Biochemistry, Brown University, Providence, Rhode Island, USA; 17 Department of Psychiatry and Human Behavior, Brown University, Providence, Rhode Island, USA; 18 Howard Hughes Medical Institute and Division of Genetics, Children's Hospital Boston, and Neurology and Pediatrics, Harvard Medical School Center for Life Sciences, Boston, Massachusetts, USA; 19 Department of Molecular Physiology & Biophysics, Center for Molecular Neuroscience, Vanderbilt University, Nashville, Tennessee, USA; 20 Institute for Juvenile Research, Department of Psychiatry, University of Illinois at Chicago, Chicago, Illinois, USA; 21 Department of Statistics, Carnegie Mellon University, Pittsburgh, Pennsylvania, USA

**Keywords:** Narrow-sense heritability, Multiplex, Simplex, Quantitative genetics

## Abstract

**Background:**

Autism spectrum disorders (ASD) are early onset neurodevelopmental syndromes typified by impairments in reciprocal social interaction and communication, accompanied by restricted and repetitive behaviors. While rare and especially de novo genetic variation are known to affect liability, whether common genetic polymorphism plays a substantial role is an open question and the relative contribution of genes and environment is contentious. It is probable that the relative contributions of rare and common variation, as well as environment, differs between ASD families having only a single affected individual (simplex) versus multiplex families who have two or more affected individuals.

**Methods:**

By using quantitative genetics techniques and the contrast of ASD subjects to controls, we estimate what portion of liability can be explained by additive genetic effects, known as narrow-sense heritability. We evaluate relatives of ASD subjects using the same methods to evaluate the assumptions of the additive model and partition families by simplex/multiplex status to determine how heritability changes with status.

**Results:**

By analyzing common variation throughout the genome, we show that common genetic polymorphism exerts substantial additive genetic effects on ASD liability and that simplex/multiplex family status has an impact on the identified composition of that risk. As a fraction of the total variation in liability, the estimated narrow-sense heritability exceeds 60% for ASD individuals from multiplex families and is approximately 40% for simplex families. By analyzing parents, unaffected siblings and alleles not transmitted from parents to their affected children, we conclude that the data for simplex ASD families follow the expectation for additive models closely. The data from multiplex families deviate somewhat from an additive model, possibly due to parental assortative mating.

**Conclusions:**

Our results, when viewed in the context of results from genome-wide association studies, demonstrate that a myriad of common variants of very small effect impacts ASD liability.

## Background

Beliefs about the genetic architecture of autism spectrum disorders (ASD) have changed dramatically over the past few decades. Early twin studies produced heritability estimates approaching 90% [1,2] and, while no specific risk loci were known at the time, it was believed that liability was conferred by a handful of genes of large effect. Later, data on the distribution of ASD within families, together with results from linkage analyses, were interpreted to mean that liability arose from many genes [3]. Recent work has definitively demonstrated the substantial contribution of *de novo* variation [4-11]. Indeed multiple studies of rare single nucleotide and copy number variants (CNVs) have suggested that 15% or more of liability traces to *de novo* mutation, effects that are genetic but not inherited [2].

Importantly, despite notable recent successes in gene discovery efforts, key questions remain regarding the overall nature and scale of the genetic contribution to ASD liability. For example, the contribution of genetics is still debated: a recent large-scale twin study [12] estimated only 38% of liability was accounted for by additive genetic effects, while common environmental factors accounted for 55% of the variance; whereas most studies of twins find much higher heritability, including studies of phenotypes in the broader spectrum (see [13,14] for review). Moreover, despite a near-consensus that common and transmitted variation must confer liability, multiple genome-wide association studies have so far not revealed replicable common polymorphisms [15] associated with ASD, and studies of rare structural and sequence mutations have largely failed to account for the anticipated risk associated with transmitted variation [6,7]. Finally, since the earliest CNV studies in ASD, it has been postulated that the architecture of simplex and multiplex autism would be strikingly different [4]. However not all studies have found marked disparities in the rate of *de novo* mutation in simplex versus multiplex families, and large effect *de novo* mutations have been characterized in both multiplex and simplex families [9,16].

Consequently, to gain insight into the broad questions regarding the nature of the genetic factors underlying ASD, we have estimated how much of the population variability in liability can be traced to inherited variation, specifically the narrow-sense heritability of ASD. Yang et al. [17] proposed elegant methods in which the heritability of liability can be estimated as a function of the covariance between trait values, in this instance affection status [18], and the genome wide genetics of the subjects. This contrasts with the usual approach of estimating heritability from the distribution of trait values in pedigrees. In the present study, these methods are applied to two ASD data sets, one from the Simons Simplex Collection (SSC) [19] and the other from the Autism Genome Project (AGP) [20]. Importantly the analysis of these two cohorts allows for an estimate of the heritability of ASD in simplex versus multiplex families as well as an assessment of how well the data fit predictions for an additive model of inheritance [21]. When all risk variation acts additively, for example, and no other forces alter the covariance of relatives, the liability for relatives of an affected individual consistently halves for each degree of separation from the proband. Therefore, we also evaluate heritability tracing to SSC and AGP parents and SSC unaffected siblings, evaluating the empirical results against simulation-derived expectations. Finally we use the same techniques to ask what residual heritability is contained in what the field calls pseudo-controls, which are genotypes formed from the alleles that parents did not transmit to their affected offspring.

## Methods

### ASD families

DNA samples from SSC and AGP family members genotyped on the Illumina Infinium^®^ 1Mv3 (duo) microarray or the Illumina Infinium^®^ 1Mv1 microarray were analyzed here. Specifically qualifying SSC samples were genotyped on the Illumina Infinium^®^ 1Mv3 (duo) microarray (71.8%) while most AGP samples were genotyped on the Illumina Infinium^®^ 1Mv1 microarray (98.7%). Both arrays genotype roughly 1,000,000 single nucleotide polymorphisms (SNPs) and the overlap between the SNP sets is almost perfect.

The SSC sample [19] includes >2,000 genotyped families. However, our analyses targeted a homogeneous subset of these data. First, we included only samples genotyped on an Illumina 1M array; families had to be ‘quads’ consisting of an unaffected mother and father, an affected proband and an unaffected sibling; and all members of a quad had to have complete genotypes (>95% completion rate). Only samples of European ancestry were included. European ancestry for the SSC families was determined using GemTools [22,23] for all available SSC probands. To conduct the ancestry analysis we selected 5,156 SNPs with at least 99.9% calls for genotypes, had minor allele frequency MAF >0.05, and were at least 0.5 Mb apart. Individuals were clustered into nine ancestry groups based on four significant dimensions of ancestry. The central five clusters, which held a total of 1,686 families, were identified as being of European descent. The ancestry cluster information combined with complete genotype information yielded a total of 965 SSC families for the analysis.

The AGP Stage 1 dataset [16,20] comprised 1,471 families, of which 1,141 were previously identified to be of European ancestry [20]. European ancestry was confirmed by analyses identical to those applied to the SSC families (see Additional file 1: Figure S1).

### Clinical evaluation

Probands for the SSC and AGP cohorts were diagnosed in a similar manner (for diagnostic protocol for SSC, see [19]; for AGP, see [16,20]). All SSC parents were screened for Autism Spectrum Disorder by the Broad Autism Phenotype Questionnaire [24] (self-report) and the Social Reciprocity Scale - Adult Research Version [25] (informant report). Moreover, family history evaluation excluded first-, second-, or third-degree relatives who met diagnostic criteria for ASD or intellectual disability. For AGP families 46.2% were known to be multiplex, another 38.2% were identified as simplex on the basis of a family history indicating no known first- to third-degree relatives with ASD, and the remaining 15.6% were of unknown status. Note that most AGP parents were not systematically evaluated for ASD, unlike those from the SSC, and when AGP parents were systematically evaluated, the results were not used to screen out affected individuals and thus multiplex families. In addition, while all available SSC family members were genotyped, only parent-proband trios were genotyped for the AGP even when additional siblings were available.

### Control subjects

Controls derived from a convenience sample, specifically 1,663 individuals from HealthABC [26]. Control samples were also genotyped on the Illumina Infinium^®^ 1Mv3 (duo) array, like most of the AGP data, providing excellent comparability with the case dataset. Moreover, we reasoned that ASD is sufficiently rare (approximately 1% [27]) that screened and unscreened controls would yield similar results.

### Filtering

To make heritability estimates comparable, we filtered all families and control subjects based on the following criteria: all were of European descent as determined by genetically-estimated ancestry (Additional file 1: Figure S1); genotypes for all family members met stringent quality control (QC) criteria; and control samples met identical QC criteria.

For the three data sets we first chose SNPs genotyped on all platforms. Then ambiguous AT, TA, CG, and GC SNPs were removed. A total of 813,960 SNP across the 22 autosomes and chromosome X were included for further quality evaluation. At the level of individuals, we required that genotyping completion rate be greater than 98%, that there be no discrepancy regarding nominal and genotype-inferred sex, and no individuals in different families were closely related. At the level of individual SNPs, each SNP must have a genotype completion rate > 98%, have MAF > 0.01, and produce a *P*-value for Hardy-Weinberg equilibrium > 0.005. Following these QC steps, data from 965 SSC quad families and 1,141 AGP families were analyzed using genotypes from 713,259 SNPs.

### Statistical calculations and motivation

#### Estimating heritability as a case-control contrast

Heritability of ASD from probands versus controls was estimated using GCTA software [28], which encodes the theory laid out in [17,18]. Prevalence of ASD was taken to be 1% [27]. For each of the analyses, Genetic Relationship Matrices (GRM) were determined for each of the 23 chromosomes using the --make-grm option in GCTA [28]. These were then combined in an overall matrix, using the --mgrm option in GCTA. The first 10 principal components of ancestry were determined using --pca in GCTA. These 10 PCA were then used as covariates for estimating the heritability using --reml in GCTA. A prevalence of 0.01 for autism spectrum disorders was used to transform the heritability on the observed scale to the heritability on the liability scale.

#### The logic of estimating heritability from unaffected family members

Due to the screening of SSC samples, no SSC parents would meet criteria for ASD. Given that is the case, what is the justification for assigning them to be affected and contrasting them to controls to estimate the heritability in the parental generation? Under the additive heritability model parents transmit many genetic variants of small effect to their offspring, with the expectation that half would be transmitted from each parent. The parents of probands are thus more similar at liability loci than expected by chance, and our goal is to estimate this increased genetic similarity. Calling the parents affected and contrasting their genotypes to that of controls is a natural approach to estimating their genetic contribution to liability and it has precedence in quantitative genetics, such as estimation of the heritability of milk production from its covariance arising from bulls, when only the bull’s female progeny give milk (for example [29]).

A similar argument follows for unaffected siblings from SSC families. These siblings should receive a random sample of the parent’s genomes and, in expectation, this sampling would include half the liability alleles carried by each parent. Thus the unaffected offspring should mirror the average liability carried by the parents and this level can be estimated by calling them affected and contrasting their genotypes to those from controls.

#### Simulations to compute expected heritability for parents and pseudo-controls

While the literature contains numerous references to the burden of risk variants carried by parents of simplex versus multiplex families, we could not find quantitative genetics analyses of it as a function of ascertainment (there is related work on the impact of multi-locus inheritance on the power of candidate gene association studies [30,31]). We therefore evaluated the *expected* heritability for parents, unaffected siblings, and pseudo-controls on the basis of simulations and the theory of quantitative genetics regarding the selection differential (for ASD, approximately 1%) and the response to selection (expected change in the population’s mean liability). The simulations are designed to mimic ascertainment for simplex and multiplex families.

One thousand SNPs having an impact on liability were simulated. The allele frequency for SNP *i*, *p*_*i*_, varied between 0.01 and 0.99. Overall heritability *h*^*2*^ across all *n* = 1000 SNPs was set to be either 0.50 or 0.75 for probands with ASD. The relative importance of each SNP, *w*_*i*_, was determined by first selecting a fraction t_*i*_ between 0 and 1 at random using a uniform distribution. These 1000 values were added to obtain *T*, and each SNP was weighted by *w*_*i*_ = t_*i*_/T. The allele substitution effect for each SNP i was then determined as $a_{i}=\sqrt{\frac{w_{i}h_{i}^{2}}{2p_{i}\left(1-p_{i}\right)}}$. For each simulation 1000 families were generated consisting of a father, mother, and one child (AGP simplex) or two children (SSC simplex or AGP multiplex). Genotypes for the parents were assigned at random using the allele frequencies, while children received alleles from the parents using the rules of Mendelian inheritance. Likewise a pseudo-control was generated by comparing the genotype of the parents to that of the proband and assigning the un-transmitted allele of each parent as the alleles for the pseudo-control’s genotype. After all genotypes in a family were assigned, the genetic contribution to the underlying liability phenotype for each individual j in the family was determined by *G*_*j*_ = ∑ _*i* = 1_^*n*^*x*_*i*_*a*_*i*_ − *μ*_*G*_ in which *x*_*i*_ is the allele count for SNP i and *μ*_*G*_ = ∑ _*i* = 1_^*n*^*p*_*i*_(1 − *p*_*i*_)*a*_*i*_ is the average genetic contribution over all genotypes. To simulate the environmental influence on the phenotype of individual j, e_j_, we drew a random number from a normal distribution with mean 0 and variance (1-*h*^*2*^). The liability phenotype was then determined as *y*_*j*_^*u*^ = *G*_*j*_ + *e*_*j*_. Affection status was then assigned based on $affection\;status=\{\begin{array}{c} not\;affected\;when\;y_{i}^{u}\;<\;2.326\\ affected\;when\;y_{i}^{u}\;\geq \;2.326 \end{array}$ representing a disease risk of 1% in the population.

Four different scenarios were simulated:

1. Primary child in the family is affected (proband), and father, mother, and designated sibling were not-affected (SSC family);

2. Proband is affected, no restriction on the other individuals in the family (unscreened simplex family);

3. Proband and second child are both affected, no restriction on the other individuals in the family (unscreened multiplex family);

4. A mixture of 60% unscreened simplex families and 40% unscreened multiplex families.

By using rejection sampling, a total of 1000 families were generated for each scenario and this procedure was repeated 100 times per scenario and proband heritability (50 and 75%). To obtain the heritability estimates for the family members, the average phenotype of the primary probands on the liability scale (S) were compared to the average phenotype of the family member of interest on the liability scale (R). The heritability estimate based on the family member was estimated as ${\overset{\wedge }{h}}^{2}=\frac{R}{S}$. Note that we also checked the heritability estimated from the probands as a function of the reduction in genetic variance in the selected group. For probands, estimated heritability was always close to 50% when that was the desired heritability and always close to 75% when that was the desired heritability.

From theoretical considerations we expected assortative mating to elevate the expected liability of pseudo-controls and evaluated its impact by a simple experiment using the simulation structure just described. Rather than randomly assign genotypes to mates, we first randomly chose the paternal genotypes at the 1,000 liability SNPs, then assigned maternal genotypes on the basis of the toss of a fair coin: heads the genotype was chosen at random, tails it was taken to be the father’s genotype. All simulations procedures were as described above, except we conducted two simulations: for simulation (a) the heritability of probands from simplex families was taken to be 50% and ascertainment followed scenario 2 above; and for simulation (b) the heritability of probands from multiplex families was set to 75% and ascertainment followed scenario 3 above.

### Robustness of results

To evaluate the robustness of the results, 1,986 individuals of European descent from the Neurogenetics Research Consortium [32] (NGRC) were available through dbGap [33] and used as a second control sample. For the NGRC study, genotypes were produced using the Illumina Infinium^®^ Human Omni2.5 microarray. Therefore, to combine all four data sets, we performed QC on 444,200 SNPs genotyped on all platforms, yielding 391,425 SNPs for analyses.

### Assessing the potential for experimental bias

To explore the impact of different cohorts and genotyping protocols on estimated heritability, we conducted a series of contrasts between SSC and AGP samples of the same relationship type – contrasting probands, mothers, fathers, and pseudo-controls – as well as HealthABC versus NGRC controls.

### Determining genomic coverage

While 713,259 SNPs were used for primary analyses, they constitute a small fraction of the SNPs in the human genome. Hence the heritability presented could underestimate total heritability. On the other hand, because genotypes of SNPs in close proximity tend to be correlated due to linkage disequilibrium, it does not follow that the coverage of the genome by the SNPs used here estimate only a small fraction of the heritability. To determine the shortfall in “genomic coverage” and how it impacts estimates of heritability, we performed an experiment using data from the 1,000 Genomes project [34], under the assumption that coverage of common variants in the 1,000 Genomes data is perfect. Assessing all SNPs genotyped in our data, as well as subsets thereof, we estimated heritability of liability. Using the same subsets, but in 1,000 Genomes subjects, we estimated levels of genomic coverage. We can then relate estimated heritability to genomic coverage to develop a functional relationship between the two.

We performed the experiment assessing “genomic coverage” as follows. We assumed genomic coverage of SNPs with MAF > 0.1 would be essentially complete for the 379 European samples analyzed by the 1,000 Genomes project. From these genomes we selected 50 1Mb regions in which at least 500 SNPs in the 1,000 Genomes samples had MAF > 0.10. Coverage of these regions by the 713,259 SNPs was calculated as a function of the number of other SNPs with MAF > 0.1 that were tagged by (correlated with) them; call the set of *M* = 713,259 SNPs “tagSNP”. The tagging evaluation was implemented using Hclust [35]. Forcing tagSNP to be in the set of selected tag SNPs from the region, Hclust evaluated how many more independent SNPs *N* were required to cover the region when the minimum linkage disequilibrium [36] r^2^ amongst tags could be no less than *X*, where *X* = {0.5, 0.7, and 0.9}. Then, for each value of *X*, M/(M+N) estimates the coverage. Next we randomly sampled 50, 25 and 12.5% of the 713,259 SNPs (356,630, 178,315, and 89,158 SNPs respectively) five times and each time estimated coverage for these subsets.

### Human subjects research statement

The research described here is in compliance with the Helsinki Declaration, including appropriate informed consent or assent [16,19,20,26,32,33].

## Results and discussion

### Estimates of heritability (h^2^)

**Figure 1:**
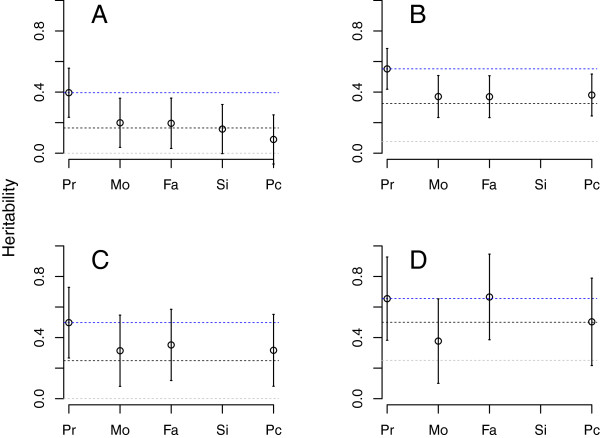
**Estimated heritability for Autism Spectrum Disorders from ASD probands (Pr), as well as for their mothers (Mo), fathers (Fa), siblings (Si) and pseudo-controls (Pc).** Blue dotted reference line is set to the estimated heritability from probands; the black line marks the *expected* heritability for first degree relatives; and the gray line marks the expected heritability from pseudo-controls. Expected values derived from simulations mimicking the recruitment strategy producing the samples for (**A**)-(**D**). (**A**) Simons Simplex Collection or SSC data; (**B**) Autism Genome Project or AGP data; (**C**) AGP data, only simplex families; (**D**) AGP data, only multiplex families.

Heritability of SSC probands, measured against HealthABC controls, was found to be 39.6% (Figure 1A, Table 1). SSC mothers, fathers and siblings, when contrasted to controls, yielded an estimated heritability approximately half that of probands (Figure 1A, Table 1), consistent with expected values from theoretical analyses of an additive model (Figure 1A). We also generate a “pseudo-control” from the alleles that parents did not transmit to their affected offspring by using the program Plink [37]. When these pseudo-controls were contrasted to the unrelated control sample they produce estimates roughly one-quarter of that identified in probands and close to the theoretical expectation, zero (Figure 1A), demonstrating that the probands received the majority of risk alleles carried by parents.

**Table 1:** **Heritability estimates and their standard errors** (**se**) **based on contrasts to HealthABC controls using genotypes from 713**,**259 SNPs**

	**SSC**		**AGP**					
	**Simplex**		**All**		**Simplex**		**Multiplex**	
	**Estimate**	**se**	**Estimate**	**se**	**Estimate**	**se**	**Estimate**	**se**
Probands	0.396	0.082	0.552	0.068	0.498	0.118	0.655	0.139
Mothers	0.199	0.082	0.371	0.070	0.314	0.119	0.377	0.141
Fathers	0.196	0.084	0.370	0.070	0.352	0.119	0.666	0.143
Siblings	0.158	0.082	--	--	--	--	--	--
Pseudo controls	0.090	0.082	0.381	0.070	0.317	0.120	0.503	0.146

When heritability is estimated using AGP probands (Figure 1B, Table 1), the point estimates are larger than those from SSC (h^2^=55.2% versus 39.6%) although the 95% confidence intervals overlap. Moreover the decline in heritability for AGP parents relative to probands is 30% (55% for probands, 37% for parents), instead of the 50% seen for SSC, and heritability estimated from pseudo-controls is also higher (38%), consistent with parental values (Figure 1B, Table 1). These results suggest that AGP parents carry a greater load of additive risk variants than SSC parents and thus are, on average, closer to the threshold of being affected.

A major difference between the SSC and AGP samples was the ascertainment and assessment process. SSC parents were systematically screened on two instruments to ensure they did not meet criteria for a spectrum diagnosis. Most parents from AGP families were not evaluated in this way, and a small fraction of those parents met criteria for ASD [9,16]. While not as systematic as the SSC phenotyping assessment, most AGP families did have available information about simplex versus multiplex status. Consequently, we were able to compare heritability of probands from AGP multiplex versus simplex families (Figure 1D, Table 2). The former was estimated at 65.5% by comparison to HealthABC, whereas probands for AGP simplex families it was 49.8%. Thus estimates of heritability for AGP simplex probands are somewhat closer to those from SSC probands (Figure 1C) than to estimates for AGP multiplex probands. Moreover, for multiplex families and the mixed set of AGP families (simplex/multiplex/unknown), both the observed and expected heritability for first-degree relatives was higher than that seen in simplex families (Figure 1). These results comport with the literature showing that unaffected relatives from multiplex families tend to exhibit more features of the broader autism phenotype than relatives in simplex families [38-40] (see Additional file 2: Table S1 for estimates from combined simplex samples).

**Table 2:** **Heritability estimates and their standard errors** (**se**) **based on contrasts to HealthABC and NGRC controls using genotypes from 391**,**425 SNPs**

	**SSC**				**AGP**			
	**HealthABC**		**NGRC**		**HealthABC**		**NGRC**	
	**Estimate**	**se**	**Estimate**	**se**	**Estimate**	**se**	**Estimate**	**se**
Probands	0.395	0.082	0.378	0.073	0.553	0.068	0.586	0.063
Mothers	0.200	0.082	0.232	0.074	0.371	0.070	0.342	0.065
Fathers	0.196	0.084	0.153	0.073	0.373	0.070	0.518	0.063
Siblings	0.158	0.082	0.170	0.073	--	--	--	--
Pseudo controls	0.090	0.082	0.107	0.073	0.380	0.070	0.446	0.065

A curious observation from AGP multiplex families was that fathers generate larger heritability than mothers. We reasoned that this could be explained by three plausible hypotheses: (1) the confidence intervals of the paternal and maternal estimates overlap, so there is no true difference; (2) the load of risk variants is, in fact, greater for AGP fathers; or (3) fathers carry a larger number of both liability and protective alleles. The last of these requires some elaboration. Males are at much greater risk for ASD than females (4:1 or greater) and parents carry additive risk factors, yet AGP fathers and mothers are largely unaffected. It is possible, then, that the increased allele sharing in unaffected fathers is due to a greater proportion of protective alleles, with females being resilient for some other reason (for example, estrogen/testosterone balance) in the face of a similar degree of genetic risk.

Our results support either the first or second hypotheses but are not consistent with the third. The first hypothesis is impossible to rule out given the limited sample size. For the second hypothesis, if AGP fathers were simply carrying greater risk, some of those additional risk alleles would be carried by the pseudo-controls and the heritability obtained from the contrast of probands and pseudo-controls should be substantially smaller than that observed from probands *versus* controls. Indeed the values are substantially smaller: 10.9% *vs*. 39.6% for SSC; 14.5% *vs*. 55.2% for all AGP; 0.0% *vs*. 49.8% for simplex AGP, and 27.1% *vs*. 65.5% for multiplex AGP. Finally, if (3) were true, then contrasting probands to pseudo-controls would produce substantial estimates of heritability because of the differentiation induced by protective alleles, but this is not observed.

### Distribution of liability alleles in the genome

**Figure 2:**
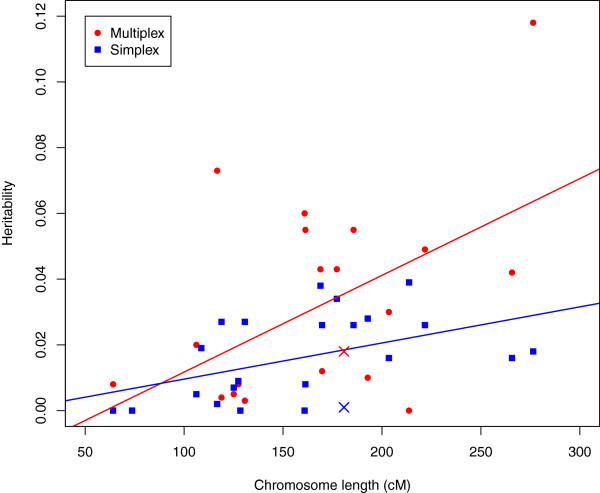
**Estimated heritability per chromosome for simplex and muliplex families.** In this figure chromosome X is marked distinctly, but each chromosome is mapped by its length.

If the additive variation for liability to ASD conforms to the traditional polygenic or infinitesimal model, then liability variants should be distributed at random over the genome. The implication is that if heritability were estimated for each chromosome, the resulting estimates should be correlated with the lengths of the chromosomes. On the other hand, if the heritability traced to a relatively small number of variants, even a few dozen, such a correlation would be unlikely. In fact, we observe significant correlation between per-chromosome heritability and chromosome length (Figure 2), both for simplex (r = 0.46, *P* value = 0.028) and multiplex (r= 0.54, *P* value = 0.0075) families.

In Figure 2 the deviation from prediction for chromosome X is surprising. For both multiplex and simplex families, heritability estimated from X is less than that predicted by its size. This is noteworthy because chromosome X has been cited as a possible source of sex-differential liability for ASD [41]. Our results suggest that common variants affecting liability do not cluster on chromosome X.

### Evaluating robustness of results

To evaluate the robustness of our results, we first contrasted the genotypes of SSC and AGP probands to a second large set of controls, 1,986 individuals from the Neurogenetics Research Consortium [32,33]. These samples, genotyped on the Illumina Infinium^®^ Human Omni2.5, were filtered and subjected to QC in an identical fashion to the HealthABC control set. There was excellent agreement of heritability estimates for ASD from the two control samples (Tables 2 and 3) despite differences in ascertainment of the controls and the different genotyping platforms.

**Table 3:** **Heritability estimates and their standard errors** (**se**) **based on contrasts to HealthABC and NGRC controls using genotypes from 391,425 SNPs but separating the AGP data into multiplex and simplex families for estimation**

	**AGP multiplex**				**AGP simplex**			
	**HealthABC**		**NGRC**		**HealthABC**		**NGRC**	
	**Estimate**	**se**	**Estimate**	**se**	**Estimate**	**se**	**Estimate**	**se**
Probands	0.650	0.139	0.710	0.140	0.503	0.117	0.494	0.114
Mothers	0.369	0.141	0.387	0.136	0.311	0.119	0.268	0.117
Fathers	0.664	0.143	0.693	0.140	0.359	0.119	0.520	0.113
Pseudo controls	0.497	0.146	0.524	0.140	0.323	0.120	0.438	0.117

Next, the impact of different cohorts and genotyping platforms on estimates of heritability was explored by conducting a series of contrasts between SSC and AGP samples of the same relationship type: contrasting probands, mothers, fathers, and pseudo-controls. Note that most SSC samples were genotyped on the Illumina^®^ 1Mv3 (duo) microarray (71.8%) while most AGP samples were genotyped on the Illumina Infinium^®^ 1Mv1 microarray (98.7%). Contrasts between SSC and AGP samples of the same relationship type (Additional file 3: Table S2) produce estimates close to the difference between their control-based heritability. Indeed the estimates from direct contrasts were usually smaller than the difference of control-based heritability (for probands, 0.08 *vs*. 0.15 ≈ 0.552-0.396 from Table 1; for mothers, 0.11 *vs*. 0.17; for fathers, 0.19 *vs*. 0.17; and for pseudo-controls, 0.22 *vs*. 0.29). Thus these results are not consistent with effects attributable to genotyping platform or ascertainment beyond multiplex/simplex status. Implicit in these results is common genetic liability - SSC and AGP probands must share many liability variants despite their differences in ascertainment. Indeed when AGP multiplex probands are contrasted to SSC probands the resulting heritability is 0.23, quite similar to that expected by the difference in their estimated heritability (0.66 - 0.40 = 0.26); and when AGP simplex probands are contrasted to SSC probands, the resulting estimated heritability, 0.0, is below that of the difference in their estimated heritability (0.50 - 0.40 = 0.10). These results suggest that the difference between multiplex and simplex families is largely a matter of degree (see also [42]), namely the number of liability alleles carried by parents, rather than a fundamental difference in the genetic architecture [4,43].

Given the remarkable similarities of heritability estimates obtained for either set of control samples (Tables 2 and 3), one might anticipate there would be little, if any, difference between these controls. When we contrasted these control samples, however, they produced a heritability of 26.5% (Additional file 3: Table S2). Mathematically, estimates of heritability arise from a high dimensional space of allele frequencies, phenotypes and their interrelationships. Therefore even if two controls groups evoke similar estimates of ASD heritability from the same sample of probands, the controls themselves need not be close in the multidimensional space of allele frequencies. What generates the differentiation between controls is unknown. It could arise from the different genotyping platforms or from differences in ascertainment. In light of this difference, the fact that both controls sets give rise to nearly identical estimates of heritability for all proband subsets is remarkable and suggests that the similarity amongst cases overwhelms differences between the controls.

### Heritability of pseudo-controls

There remains an unexplained feature of the results: estimates of heritability for pseudo-controls tend to be elevated over their theoretical values (Figure 1). Several genetic forces could be at play. The simulations to derive the distribution of liability in families also produce estimates for pseudo-controls. Those results show (Figure 1) that while the expected heritability for simplex families is zero, multiplex status raises the expected value to 20%. It is not unreasonable to assume that the simplex collections analyzed here contain families with unrealized multiplex potential, and that might be especially true for AGP families that had ascertainment criteria less stringent than those for SSC families.

A factor that will elevate the expected heritability in pseudo-controls is positive assortative mating (henceforth assortative mating). Assortative mating on phenotypes related to ASD liability has been previously reported [39]. When parents are genetically similar at liability loci and they bear affected offspring, their gametes will tend to be highly enriched for risk alleles, even those that are not transmitted to affected offspring. Simple simulations mimicking assortative mating show that it can exert an impact similar to the difference between simplex and multiplex status. When simplex probands had heritability of 50% (that is, simulation *a* in Methods), the expected heritability of pseudo-controls was 11.3% – versus 0% without assortative mating. When multiplex probands had heritability of 75%, the expected heritability of pseudo-controls was 42.8% – versus 20.2% without assortative mating. These simple experiments were not intended to cover the range of plausible scenarios for assortative mating relevant to ASD, which would be impossible, but rather to demonstrate the effect of such mating on the nature of pseudo-controls. Thus assortative mating could be an important and salient source of enrichment. Whether these forces explain all of the elevated heritability for pseudo-controls will require further data and analyses.

### Impact of genome coverage

Because the set of SNPs used for primary analyses constitute a small fraction of the SNPs in the human genome, estimates of heritability (Figure 1) could be biased downward. Still, due to linkage disequilibrium, the degree of bias is not trivial to estimate. Therefore we performed an experiment to evaluate the shortfall in genomic coverage and how it impacts estimates of heritability. Results from the experiment are shown in Additional file 4: Figure S2, in which estimated heritability was plotted against estimated coverage. These results suggest that heritability estimates from probands, as shown in Figure 1, are good approximations. They represent only slight underestimates of what would be obtained had the entire genome been sampled.

In total our results demonstrate that a substantial portion of ASD liability arises from inherited variation acting additively. This pattern holds both for simplex and multiplex families, with the burden of liability greater in multiplex families, consistent with theoretical and empirical [38-40] results. The modeling reported here does not differentiate between additive effects due to common versus rare variation. Nonetheless it is reasonable to assume that most of the estimated heritability traces to common variants because linkage disequilibrium between the common variants analyzed and rare liability variants should, on average, be small [44]. Thus the additive contribution of rare variants to ASD liability is likely underestimated. Imperfect coverage must also have an impact, but our analyses suggest its impact is not large (Additional file 4: Figure S2).

Our analyses cannot address other features of the genetic architecture of ASD, including non-additive genetic effects, which add to ASD’s broad-sense heritability [45], and de novo mutations. In addition, because they underestimate the impact of rare inherited variation, they differ from family-based estimates, such as from twin studies, that do capture these effects. Still our findings of substantial heritability are consistent with the majority of twin studies [1,2] and are richer in some ways because the analytic technique [17,18] used here provides a direct estimate of the proportion of liability attributable to additive genetic effects, whereas twin studies obtain their estimates by relying on assumptions that are approximations. For example, Zuk et al. [45] point out that non-additive genetic effects are almost surely a component of the genetic architecture of any trait, but these effects cannot be captured by twin designs. Yet for autism and other psychiatric disorders non-additive genetic effects could be an integral component [46-48]. Twin designs also fail to capture other features, such as maternal effects [49] and de novo mutations, which are an important component of ASD genetic architecture [4-11].

A recent ASD twin study [12] estimates 38% of ASD liability traces to additive genetic effects while 55% traces to common environment. Our point estimates would be close to theirs if ascertainment of their families was like that for SSC families, but not like that for AGP families. A substantial fraction of their dizygotic twins, however, are multiplex for ASD. Thus their point estimate for heritability from additive genetic effects is low relative to ours. If rare inherited variation contributes substantially to liability for ASD, this makes the 38% estimate seem lower still because twin studies should capture these effects whereas our estimates cannot.

Genomewide association studies [18,50-52] have detected only a handful of SNPs, all of small effect and none replicating reliably. Teaming this observation with our estimates of heritability (Figure 1) and the fact that these studies are underpowered to detect genetic variants of small effect size, but are otherwise well powered [15], we conclude there must be thousands of SNPs scattered across the genome with common liability alleles. Analyses of chromosome-specific heritability support this conclusion (Figure 2). Employing analyses like those proposed by Stahl et al. [53] could estimate this distribution of effects.

Because these loci have small effect, samples far larger than exist today will be required to identify a substantial fraction of them using standard genome-wide association methodology. Hence, for the immediate future, ample “missing heritability” for ASD will remain. Ingenious designs will be required in the near term [54] to identify SNPs affecting liability. In the longer term GWAS of a large number of ASD subjects, at least on the order of that performed for schizophrenia [55-57], should be one of the priorities for the field of ASD genetics.

One way forward is to exploit shared liability across psychiatric disorders, taking advantage of larger samples [58] afforded by cross-disorder meta-analysis. There is now sound evidence for common variants affecting liability for schizophrenia [55-57], including a study similar to ours [46]. Given the documented sharing of rare variants affecting risk for both disorders (for example [59]), it would not be surprising to find that some common variants affect liability to both schizophrenia and ASD.

The estimated heritability for schizophrenia using methods similar to ours is 23% [46]; for bipolar disorder and similar methods it is 40% [60]; and for major depression it is 32% [61]. None of these studies separate out simplex and multiplex families, so in that sense they are most comparable to the estimate obtained over all AGP families, 55%, although the representation of multiplex families in the AGP sample is likely larger than for the other samples. Regardless of the differences in simplex/multiplex representation, these estimates are stochastically similar, in view of their standard errors, emphasizing that common variants affect liability for most if not all psychiatric disorders. Moreover their impact appears to be similar in magnitude across disorders, as measured by heritability estimated from common variants.

That ASD shows the largest estimated heritability is notable and could reflect the fact that the sibling recurrence risk is, on average, higher for siblings of an ASD proband than for siblings of probands diagnosed with schizophrenia, bipolar disorder or major depression. Sibling recurrence risk is a ratio, defined as the probability of a sibling being affected, given that the proband is affected, divided by the prevalence of the disorder in the general population. Recent studies put this recurrence risk at almost 20 for ASD [62], whereas for schizophrenia it is 6 to 10 fold [63], for bipolar disorder it is 4 to 10 fold [64], and for major depression it is roughly twofold [64]. The larger heritability could also trace to differences among studies. It is possible that our estimates of heritability are inflated by unknown differences between our case and control samples, including ascertainment biases and genotype quality. Regarding the latter, we selected case and control samples genotyped on the same genotyping platform to minimize differences and we did not detect any large differences in allele frequencies, but we cannot rule out subtle differences in quality.

Regarding identification of common variants affecting liability, our results suggest that the contrast of case and pseudo-control genotypes, the “family-based” analysis, is not optimal. In many samples pseudo-controls carry a substantial burden of risk variants and their presence degrades the power of family-based analysis to detect risk SNPs (see also [30,31]). Instead it appears that population-based controls contrasted with ASD cases would be a more powerful design [65], even after adjusting for ancestry [66]. In this regard it is intriguing that the earliest GWAS of ASD [50] used population-based controls to identify a single locus at 5p14.1, and this result has since garnered support from a functional study that reveals a plausible biological link to ASD liability [67].

The genetic architecture of ASD has numerous components: additive, non-additive and de novo genetic effects, as well as gene-gene and gene-environment interactions. The results shown here are relevant to only one of these components. Other components, such as de novo events, are also known to make a substantial contribution to liability [4-11], while others remain to be thoroughly investigated [45]. Already analyses of rare variation of major effect has revealed a substantial number of genes affecting liability [8-11,68-70]; it is reasonable to predict that common variants regulating expression of those ASD genes could also affect liability [71]. We hypothesize that the interplay of rare and common variants is critical not only to liability itself, but to the expression of ASD or other relevant psychiatric and developmental disorders. The dynamics of this interplay will likely be an important area for future autism research.

## Conclusions

Common genetic polymorphisms exert substantial additive genetic effects on ASD liability and their impact differs by ascertainment strategies used to recruit families. For simplex families, who have only a single affected individual in multiple generations, approximately 40% of liability traces to additive effects whereas this narrow-sense heritability exceeds 60% for ASD individuals from multiplex families. Data for simplex ASD families follow the expectation for additive models closely. Data from multiplex families deviate somewhat from an additive model. This result is consistent with what would be expected from positive assortative mating, but our data do not prove such a pattern of mating occurred. In light of results from genome-wide association studies, there must be many common variants of very small effect affecting liability to ASD.

## Availability of supporting data

The data sets supporting the results of this article are available in the repositories: Simons Foundation Autism Research Initiative, SFARI [http://sfari.org/sfari-initiatives/simons-simplex-collection]; and the National Institutes of Health database of Genotypes and Phenotypes, dbGaP [http://www.ncbi.nlm.nih.gov/gap].

## Abbreviations

AGP: Autism Genome Project; ASD: Autism Spectrum Disorders; CNVs: Copy Number Variants; GCTA: Genome-Wide Complex Trait Analysis, Software used to estimate heritability, amongst others; GRM: Genetic Relationship Matrices; HealthABC: A sample of subjects used as controls and genotyped on the Illumina Infinium® 1Mv3 (duo) array; MAF: Minor Allele Frequency; NGRC: Neurogenetics Research Consortium, a sample of subjects used as controls and genotyped on the Illumina Infinium® Human Omni2.5 microarray; QC: Quality Control; SNPs: Single Nucleotide Polymorphisms; SSC: Simons Simplex Collection.

## Competing interests

The authors declare no competing financial interests.

## Authors’ contributions

MWS supervised the overall project, EHC its phenotypic portions; LK, KR and BD conceived of the analyses; LK implemented the analyses; EHC, KR, MWS, SJS, and BD wrote the first draft of the manuscript; all others authors commented on and refined it. Most authors recruited families, produced or evaluated data and commented on the manuscript. All authors read and approve the final manuscript.

## Supplementary Material

Additional file 1 **Figure S1.** Ancestry projects for principal component 1 (PC.1) versus principal component 2 (PC.2) for the samples used in the analysis of heritability. Red dots represent subjects with an ASD diagnosis and blue are controls. HealthABC=HABC. Click here for file

Additional file 2 **Table S1.** Heritability estimates and their standard errors (se) using 391,425 SNP when AGP and SSC simplex family data are combined or only multiplex AGP families are analyzed. Analyses include all HealthABC and NGRC control samples. Click here for file

Additional file 3 **Table S2.** Heritability estimates and their standard errors (se) obtained when contrasting AGP and SSC samples of the same relationship type, as well as contrasting HealthABC versus NGRC controls. Click here for file

Additional file 4 **Figure S2.** Heritability for ASD probands as a function of estimated “genomic coverage” for varying levels of r^2^. Coverage is estimated as the fraction of all known SNPs identified by 1000 Genomes with minor allele frequency > 0.1 tagged by the set of SNPs used to estimate heritability for probands; see Methods for more details. From the left points map onto 12.5%, 25%, 50%, and 100% of the SNPs used to estimate heritability. Top line is for probands from multiplex families, bottom for probands from simplex families. Click here for file
