# Supplementary material for: Common genetic variants, acting additively, are a major source of risk for autism
Source: Mol Autism. 2012 Oct 15;3:9. doi: 10.1186/2040-2392-3-9 (PMC3579743; doi:10.1186/2040-2392-3-9)
Supplement: Additional file 1 — Figure S1. Ancestry projects for principal component 1 (PC.1) versus principal component 2 (PC.2) for the samples used in the analysis of heritability. Red dots represent subjects with an ASD diagnosis and blue are controls. HealthABC=HABC. [file 2040-2392-3-9-S1.pdf]

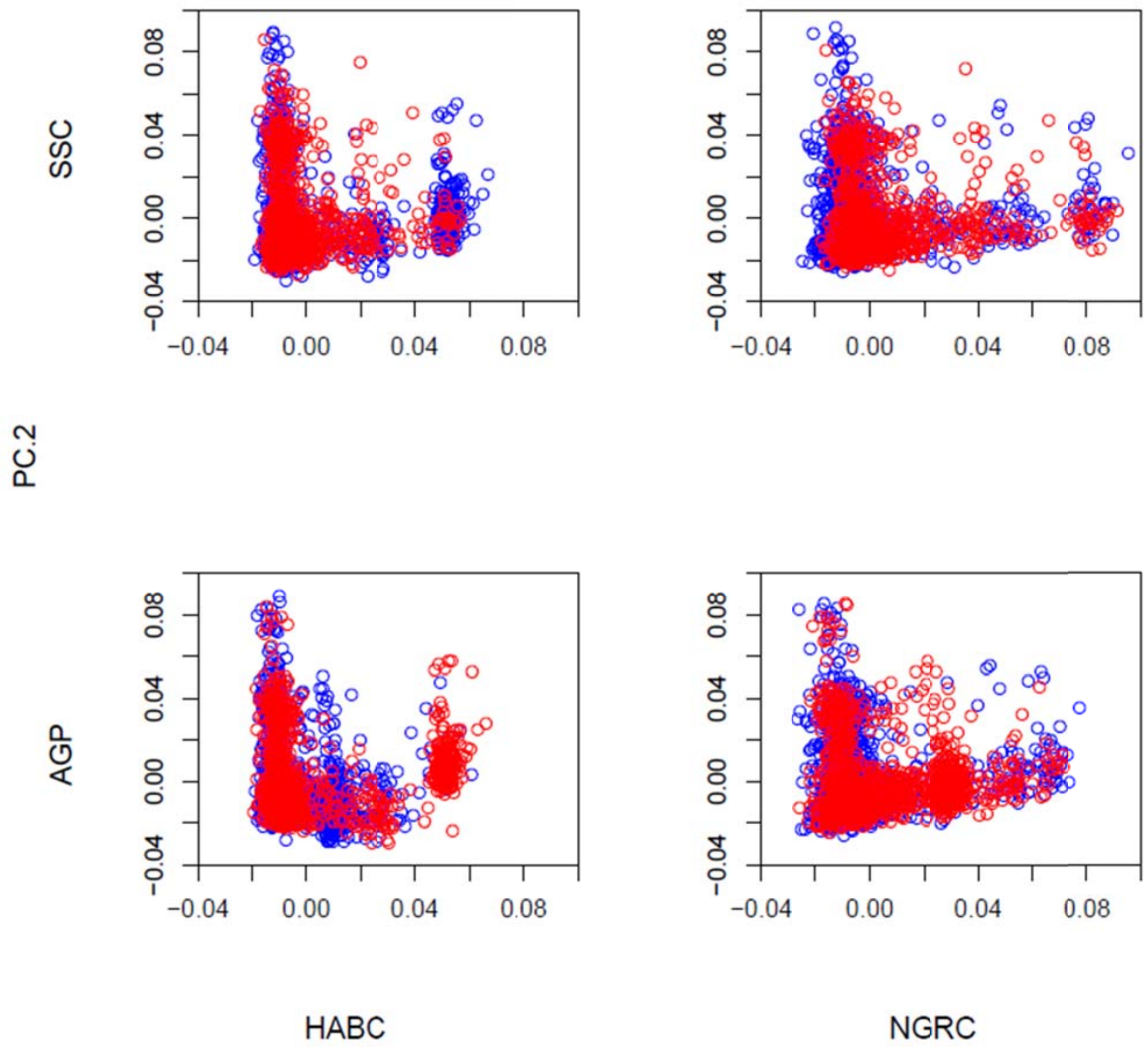

Supplementary Figure 1. Ancestry projects for principal component 1 (PC.1) versus principal component 2 (PC.2) for the samples used in the analysis of heritability. Red dots represent subjects with an ASD diagnosis and blue are controls. HealthABC = HABC.
