# Supplementary material for: Common genetic variants, acting additively, are a major source of risk for autism
Source: Mol Autism. 2012 Oct 15;3:9. doi: 10.1186/2040-2392-3-9 (PMC3579743; doi:10.1186/2040-2392-3-9)
Supplement: Additional file 2 — Table S1. Heritability estimates and their standard errors (se) using 391,425 SNP when AGP and SSC simplex family data are combined or only multiplex AGP families are analyzed. Analyses include all HealthABC and NGRC control samples. [file 2040-2392-3-9-S2.pdf]

Supplementary Table 1. Heritability estimates and their standard errors (se) using 391,425 SNP when AGP and SSC simplex family data are combined or only multiplex AGP families are analyzed. Analyses include all HealthABC and NGRC control samples.

	**Multiplex**		**Simplex**	
	**estimate**	**se**	**estimate**	**se**
Probands	0.617	0.129	0.371	0.045
Mothers	0.326	0.126	0.258	0.045
Fathers	0.609	0.131	0.266	0.045
Pseudo Controls	0.454	0.131	0.152	0.044
