# Supplementary material for: Common genetic variants, acting additively, are a major source of risk for autism
Source: Mol Autism. 2012 Oct 15;3:9. doi: 10.1186/2040-2392-3-9 (PMC3579743; doi:10.1186/2040-2392-3-9)
Supplement: Additional file 3 — Table S2. Heritability estimates and their standard errors (se) obtained when contrasting AGP and SSC samples of the same relationship type, as well as contrasting HealthABC versus NGRC controls. [file 2040-2392-3-9-S3.pdf]

Supplementary Table 2. Heritability estimates and their standard errors (se) obtained when contrasting AGP and SSC samples of the same relationship type, as well as contrasting HealthABC versus NGRC controls.

	SSC versus All AGP		SSC versus Simplex AGP	
	**estimate**	**se**	**estimate**	**se**
Probands	0.084	0.092	0.000	0.139
Mothers	0.109	0.091	0.063	0.140
Fathers	0.188	0.089	0.126	0.129
Pseudo Controls	0.215	0.090	0.176	0.132
**Controls**				
	estimate		se	
HealthABC versus NGRC	0.265		0.071	
