# Supplementary material for: Common genetic variants, acting additively, are a major source of risk for autism
Source: Mol Autism. 2012 Oct 15;3:9. doi: 10.1186/2040-2392-3-9 (PMC3579743; doi:10.1186/2040-2392-3-9)
Supplement: Additional file 4 — Figure S2. Heritability for ASD probands as a function of estimated “genomic coverage” for varying levels of r2. Coverage is estimated as the fraction of all known SNPs identified by 1000 Genomes with minor allele frequency > 0.1 tagged by the set of SNPs used to estimate heritability for probands; see Methods for more details. From the left points map onto 12.5%, 25%, 50%, and 100% of the SNPs used to estimate heritability. Top line is for probands from multiplex families, bottom for probands from simplex families. [file 2040-2392-3-9-S4.pdf]

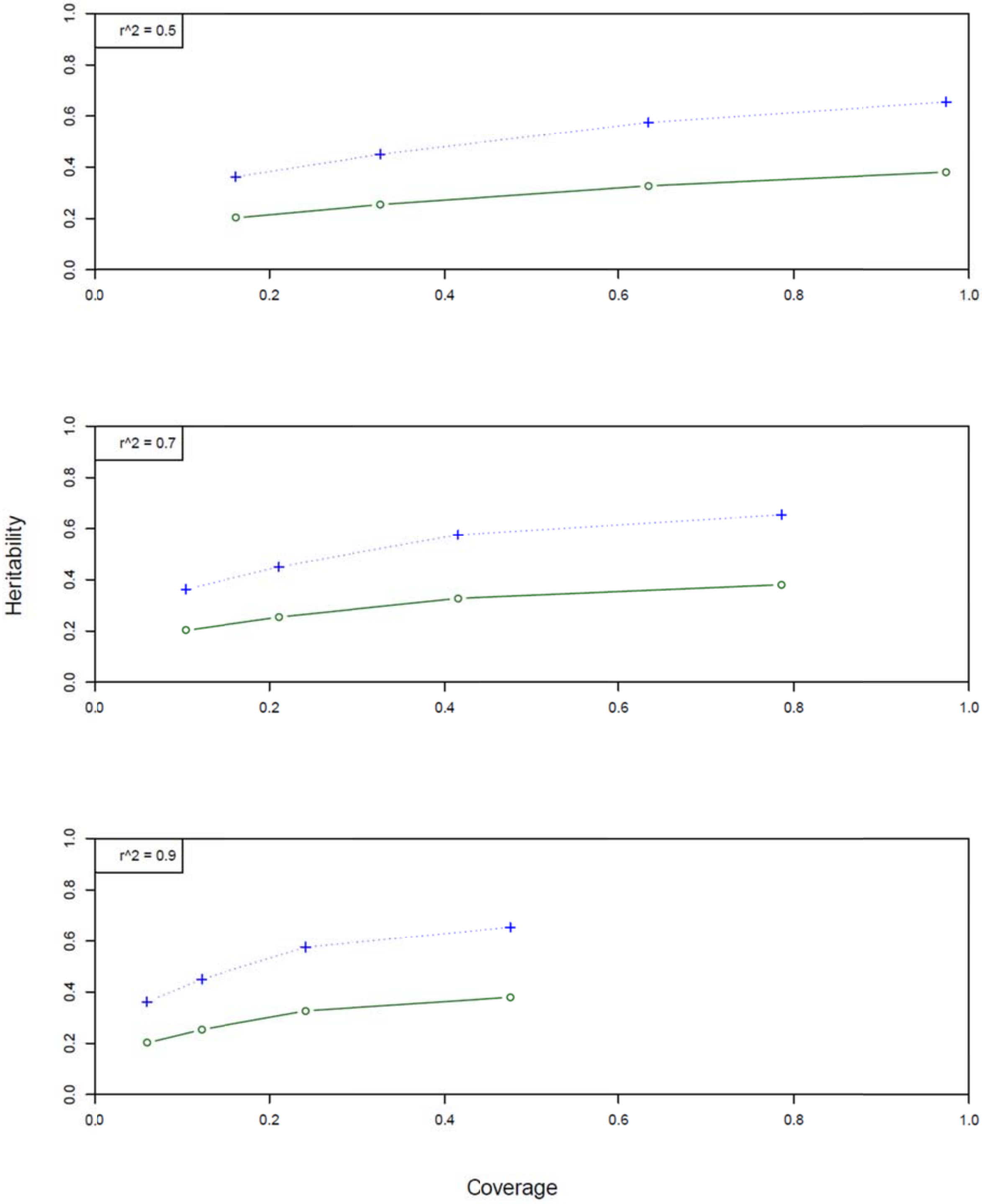

Supplementary Figure 2. Heritability for ASD probands as a function of estimated "genomic coverage" for varying levels of r^2. Coverage is estimated as the fraction of all known SNPs identified by 1000 Genomes with minor allele frequency > 0.1 tagged by the set of SNPs used to estimate heritability for probands; see Online Methods for more details. From the left points map onto 12.5%, 25%, 50%, and 100% of the SNPs used to estimate heritability. Top line is for probands from multiplex families, bottom for probands from simplex families.
